# Pathogenesis and Inhibition of Flaviviruses from a Carbohydrate Perspective

**DOI:** 10.3390/ph10020044

**Published:** 2017-05-04

**Authors:** So Young Kim, Bing Li, Robert J. Linhardt

**Affiliations:** 1 Biochemistry and Biophysics Graduate Program, Center for Biotechnology and Interdisciplinary Studies, Rensselaer Polytechnic Institute, Troy, NY 12180, USA; pinkes2@rpi.edu; 2 Guangdong Province Key Laboratory for Green Processing of Natural Products and Product Safety, Guangzhou 510640, China; 3 School of Food Science and Technology, South China University of Technology, Guangzhou 510640, China; 4 Department of Chemistry and Chemical Biology, Center for Biotechnology and Interdisciplinary Studies, Rensselaer Polytechnic Institute, Troy, NY 12180, USA; 5 Department of Biological Science, Center for Biotechnology and Interdisciplinary Studies, Rensselaer Polytechnic Institute, Troy, NY 12180, USA; 6 Department of Chemical and Biological Engineering, Center for Biotechnology and Interdisciplinary Studies, Rensselaer Polytechnic Institute, Troy, NY 12180, USA; 7 Biomedical Engineering, Center for Biotechnology and Interdisciplinary Studies, Rensselaer Polytechnic Institute, Troy, NY 12180, USA

**Keywords:** dengue virus, DC-SIGN, envelope protein, flavivirus, flavivirus inhibitors, glycosaminoglycans, Japanese encephalitis virus, proteoglycans, viral infection, West Nile virus, yellow fever virus, Zika virus

## Abstract

Flaviviruses are enveloped, positive single stranded ribonucleic acid (RNA) viruses with various routes of transmission. While the type and severity of symptoms caused by pathogenic flaviviruses vary from hemorrhagic fever to fetal abnormalities, their general mechanism of host cell entry is similar. All pathogenic flaviviruses, such as dengue virus, yellow fever virus, West Nile virus, Japanese encephalitis virus, and Zika virus, bind to glycosaminglycans (GAGs) through the putative GAG binding sites within their envelope proteins to gain access to the surface of host cells. GAGs are long, linear, anionic polysaccharides with a repeating disaccharide unit and are involved in many biological processes, such as cellular signaling, cell adhesion, and pathogenesis. Flavivirus envelope proteins are *N*-glycosylated surface proteins, which interact with C-type lectins, dendritic cell-specific intercellular adhesion molecule-3-grabbing non-integrin (DC-SIGN) through their glycans. In this review, we discuss both host and viral surface receptors that have the carbohydrate components, focusing on the surface interactions in the early stage of flavivirus entry. GAG-flavivirus envelope protein interactions as well as interactions between flavivirus envelope proteins and DC-SIGN are discussed in detail. This review also examines natural and synthetic inhibitors of flaviviruses that are carbohydrate-based or carbohydrate-targeting. Both advantages and drawbacks of these inhibitors are explored, as are potential strategies to improve their efficacy to ultimately help eradicate flavivirus infections.

## 1. Introduction

Each year, 13.4 million deaths worldwide are caused by various parasitic, bacterial, and viral infectious diseases [1]. Mosquito-borne infectious diseases annually cause several million deaths and hundreds of millions of cases [2]. Dengue virus (DENV), the world’s most dangerous mosquito-borne flavivirus (FLV) disease, places 2.5 billion at risk of infection and results in 20 million cases each year in 100 countries and to date there is no completely effective vaccine [3]. Although more than 105 million people have been vaccinated for yellow fever virus (YFV) in West Africa, 84 to 170 thousand severe cases and 29 to 60 thousand deaths were estimated in Africa during 2013 [2]. While of less risk, West Nile virus (WNV) has no approved human vaccine and outbreaks in the U.S. from 1999–2010 reminds us that pandemic of vector-borne pathogens is still accessible due to frequent importation through global travel [2]. Recently, Zika virus (ZIKV) joined the list of FLVs of concern due to its ability to cross the placental barrier and cause serious birth defects with 2500 reported congenital syndromes worldwide and nearly 4000 ZIKV infection in pregnant women in the U.S. and its territories [4,5]. DENV, YFV, WNV, ZIKV, Japanese encephalitis virus (JEV), and tick-borne encephalitis virus (TBEV) belong to the *Flaviviridae* family. FLVs are enveloped, positive single stranded RNA viruses with varying symptoms from hemorrhagic fever and fatal neurological diseases to fetal defects. There are currently no approved antivirals for treating FLVs.

**Figure 1:**
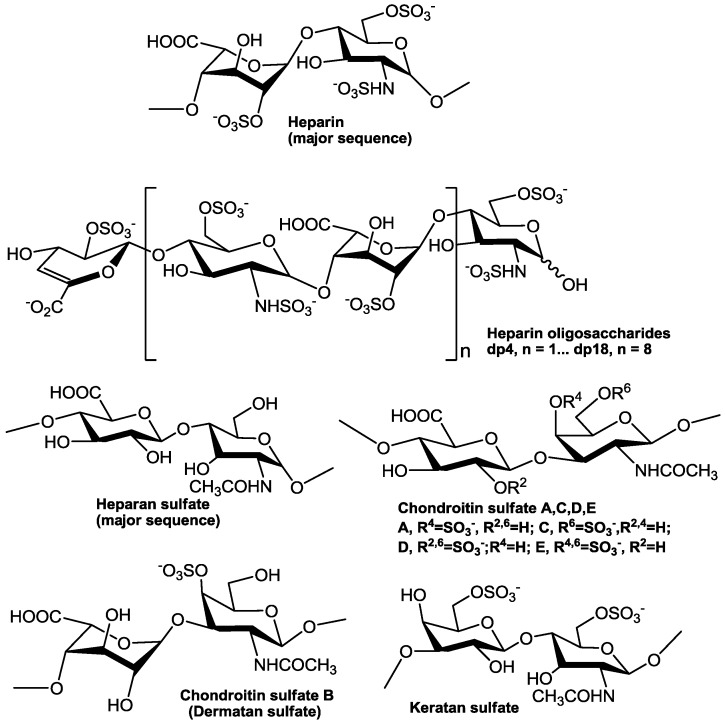
Chemical structures of glycosaminoglycans and heparin oligosaccharides.

**Figure 2:**
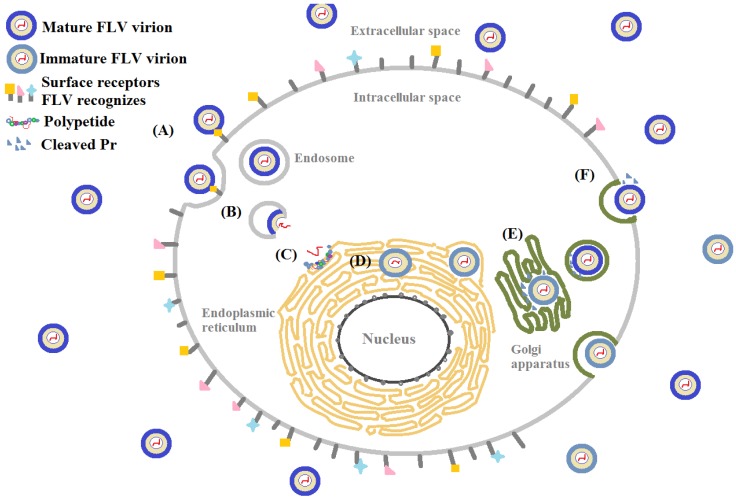
Host cell entry of flavivirus (FLV) (**A**) adsorption and (**B**) internalization confirmation change of envelope protein triggers membrane fusion and viral genome release; (**C**) replication and (**D**) translation beginning of *N*-linked glycosylation and viral assembly in the endoplasmic reticulum; (**E**) completed glycosylation and pre-membrane (prM) protein is cleaved to become a mature virion in the Golgi apparatus; (**F**) exocytosis of a mature virion.

There are many similarities in pathogenesis of FLV in host cells. Glycosaminoglycans (GAGs), for example, are the initial co-receptors that all pathogenic FLVs utilize for the infection of host cell [6,7,8,9,10,11,12,13]. GAGs are anionic, unbranched polysaccharides comprised of repeating disaccharide units located on the surface of eukaryotic cells and in their extracellular matrix (ECM; Figure 1). GAGs are involved in many biological processes, including cell adhesion, cell migration, tissue repair, ECM assembly, inflammation, and pathogenesis [14]. After successfully making contact with the host cell surface through their binding to GAGs, FLV next interact with protein-based receptors [15,16,17,18,19,20,21,22,23,24,25,26,27,28,29,30,31,32]. Finally, FLVs infiltrate into the host cell through clathrin-mediated endocytosis, accompanied by a conformation change of envelope protein and membrane fusion and release of the viral genome (Figure 2) [33,34].

This review examines the role of carbohydrates in the mechanism of FLV infection and the potential of carbohydrates in combating FLV pathogenesis. The roles of both host and viral glycans, including host cell GAGs and FLV envelope protein (FLVE) *N*-glycosylation, in FLV pathogenesis and the interactions of GAGs-FLVE and FLV envelope protein-dendritic cell-specific intercellular adhesion molecule-3-grabbing non-integrin (FLVE-DC-SIGN) will be addressed. This review will particularly focus on the viral and host surface interactions in the early stage of FLV entry. FLV inhibitors that are either glycan-based or target host or viral glycans are described. The pitfalls of currently investigated inhibitors and strategies that may improve them are discussed.

## 2. Glycan-Mediated Flavivirus Entry

**Figure 3:**
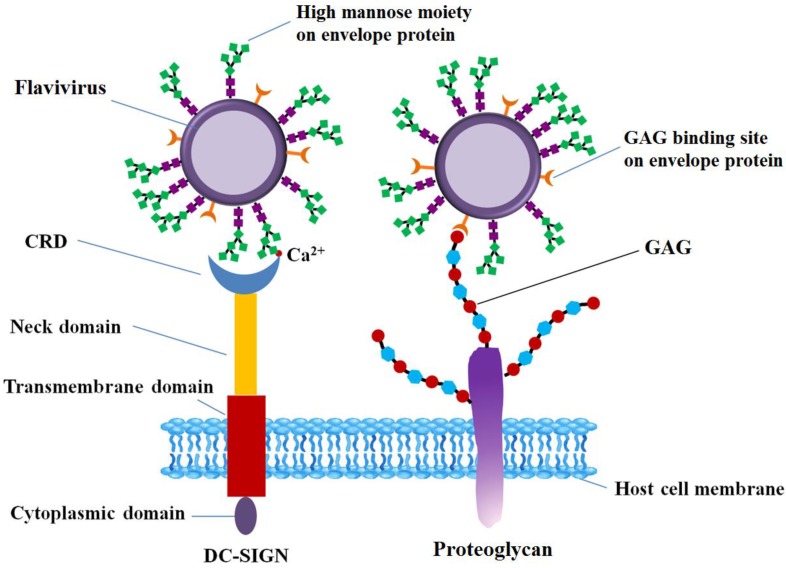
E protein binding to DC-SIGN or GAG. Carbohydrate recognition domain (CRD) of DC-SIGN with four domains (a cytoplasmic domain, a transmembrane domain, an extracellular neck domain, and a carbohydrate recognition domain) in molecular structure in the presence of Ca^2+^ recognizes the high mannose moiety on glycosylated E protein. Flavivirus binds to glycosaminoglycans (e.g., heparan sulfate or chondroitin sulfate depending on the virus) through their envelope proteins. DC-SIGN: dendritic cell-specific intercellular adhesion molecule-3-grabbing non-integrin; GAG: glycosaminoglycan.

From the perspective of not only pathogenesis but also therapy, understanding of FLVs entry into host cells with greater precision and resolution is of great importance. FLVs enter host cells through a receptor-mediated mechanism [35]. Multiple host cell receptors have been identified to facilitate FLV entry, including GAG [6,36], heat-shock proteins (heat-shock protein 70, 90, R67, R80) [15,25,37], a 45 kD mosquito glycoprotein [38], neolactotertraosylceramide [16], CD14 [17], GRP78/BiP [18], phosphatidylserine (PS) receptors such as T-cell immunoglobulin and mucin domain receptor (TIM) [39], Tyrosine-protein kinase receptor (TYRO3) [40], and AXL [41], and proto-oncogene tyrosine-protein kinase (MERTK) [42]. C-type lectins implicated in FLV entry include DC-SIGN [22,43], and the mannose receptor [23], C-type lectin domain family 5 member A (CLEC5A) [44]. Prohibitin was the first discovered receptor involved in pathogenesis of DENV in insect cells [45]. Among these receptors, GAGs, a 45 kD mosquito glycoprotein, and neolactotertraosylceramide are some of the carbohydrate-based host cell receptors. C-type lectins also involve carbohydrates as they function through their binding to glycans. Moreover, the structural glycoproteins, the envelope proteins in particular, are ligands that interact with cell surface receptors [46]. In this section, glycan-protein interactions that mediates the FLV entry into host cells on surface level, including FLVE, GAGs and C-type lectin are discussed (Figure 3).

### 2.1. The Flavivirus Envelope Proteins and Their Glycosylation

The FLV genome encodes for a single polypeptide that ultimately gets processed into the capsid (C), the membrane (prM/M), the envelope (E) protein, and seven nonstructural (NS) proteins, NS1, 2a, 2b, 3, 4a, 4b, and 5 [47,48]. The E protein mediate important processes in viral infection, such as viral attachment, fusion, penetration, hemagglutination; thus, it determines host range, cell tropism, virus virulence and attenuation [49,50]. The structures of the recombinant E proteins of TBEV [51], DENV [52], WNV [53,54], JEV [55] and ZIKV [56] have been solved by X-ray crystallography, and they exhibit great structural similarities. These E proteins consist of 500 amino acids and can be divided into three functional domains. Domain I is a central, stranded β-barrel, which contains a single *N*-linked glycan and is interspersed with domain II. Domain II is a long, finger-like protrusion from domain I, which contains the most of residues involved in dimeric interactions and houses the hydrophobic fusion peptide mediating post-entry endosomal fusion [57,58,59,60,61]. Domain III is an immunoglobulin-like fold, linked to the central domain I through a flexible stretch of amino acids, and characteristic of many cell receptors [62,63,64,65,66]. Domain III of FLVEs also contains GAG binding sites comprised of many surface basic and acidic residues that are contribute to hydrogen bonding [51,52,54,55,56]. Although the overall FLVE protein architecture is conserved, there are still significant differences within FLV family. In contrast to DENV E and TBEV E, WNV E does not form dimers in the crystal and contains more hydrogen bonds or buried surface area at the domain I-II interface [53]. An extra β strand (β5a) is formed in DENV-2 domain III, which is not found in DENV-3, DENV-4 [50]. The central domain I of JEV is composed of a nine stranded β-barrel [56], while domain I of DENV is an eight stranded β-barrel [57,59,61].

Glycosylation of FLVEs is an important determinant in the interaction of FLVs with host cells. Glycosylated variants of WNV were more virulent and had higher viremic levels in young chicks than the non-glycosylated variants [67]. The highly pathogenic WNV emerging in the late 1990s was attributed to glycosylation in WNV E. Viral replication in birds is enhanced by the glycosylation of E protein and may result in increased transmissibility among bird population and higher pathogenicity in birds [68]. Glycosylation of FLVEs takes place in the host cells that provide most or all of enzymes and saccharide substrates, since the FLVs themselves do not encode these enzymes [69,70]. During glycosylation, a pre-assembled oligosaccharide Glc_3_Man_9_GlcNAc_2_ (Glc (glucose), Man (mannose), and GlcNAc (*N*-acetylglucosamine)) is trimmed stepwise by glucosidases and mannosidases and then elaborated through the action of *N*-acetylglucosaminyltransferase, galactosyltransferases, fucosyl-transferases, and sialyltransferases [71]. FLVs derived from different vectors may have structurally different *N*-linked glycans since the enzymes involved in glycosylation produced by different vectors, such as insect and mammalian cells, are different. For example, the structures of the *N*-linked glycans on the viral glycoproteins produced by insect cells are much less complex than those produced by mammalian cells [71]. Hacker et al. [43] analyzed mosquito-derived DENV glycoproteins using endoglycosidase, PNGaseF or EndoH, and lectins, *Galanthus nivalis* agglutinin (GNA) and *Datura stramonium* agglutinin (DSA), and found they have a mix of high-Man and paucimannose glycans. Further, using both lectin microarray and matrix assisted laser desorption ionization-time of flight mass spectrometry (MALDI-TOF-MS), Lei et al. proved that *N*-glycan structure on the surface of mature DENV-2 derived from mosquito cells was highly heterogeneous [72]. Five types of *N*-linked glycans were identified comprised of Man, *N*-acetylgalactosamine (GalNAc), GlcNAc, fucose (Fuc) and sialic acid (SA). High Man-type *N*-linked oligosaccharides and galactose (Gal)-containing of *N*-linked glycans were the major structures. In viruses derived from mammalian cells, the *N*-linked glycans were a mix of high-mannose and complex type glycans [43]. The number and location of glycosylation motifs in the E vary considerably both between and within FLV species [73]. It is well known that DENV E has two potential *N*-linked glycosylation sites at Asn-67 and Asn-153 [43]. The Asn-67 site is unique to DENV E whereas Asn-153 (or nearby Asn-154) is common to other FLVs, with the exception of Kunjin virus [51,74,75]. Interestingly, DENV2 E and DENV4 E proteins are glycosylated only at the Asn-67 site while DENV1 and DENV3 E proteins are glycosylated at both Asn-67 and Asn-153 sites [74]. *N*-linked glycosylation at Asn-67 (or nearby Asn-64) is critical for the survival of the virus in either mammalian or insect cell culture [76]. The high-mannose *N*-linked glycan at Asn-154 of JEV E was shown to be crucial for JEV binding to DC-SIGN [77]. The glycosylation site in WNV E was also confirmed at Asn-154 [49].

### 2.2. Glycans on Host Cells Mediating Flavivirus Entry

#### 2.2.1. Flavivirus Binding to Glycosaminoglycans

GAGs are linear and negatively charged polysaccharides that have molecular weights of 10–100 kDa; and they contain repeating disaccharide units of uronic acid (D-glucuronic (GlcA) acid or L-iduronic acid (IdoA)) and hexosamine (D-GalN or D-GlcN) (Figure 1). Hence, GAGs differ according to the type of hexosamine, hexose or hexuronic acid unit that they contain, as well as the chirality and position of the glycosidic linkage between their saccharide units [78]. Heparan sulfate proteoglycans (HSPGs) are sulfated GAGs that represent a major GAGs component of the cell surface of eukaryotes [8]. HSPGs have the common characteristic of containing one or more covalently attached heparan sulfate (HS) chain [79]. The HS GAG has the major repeating unit of monosulfated →4)GlcNAc(1→4)GlcA/IdoA(1→ per disaccharide [80,81]. HS contains a considerable number of negatively charged sulfo and carboxyl groups that can interact with numerous proteins that carry positive charges including growth factors, morphogens, ECM proteins and pathogen surface proteins [6,82,83,84,85,86]. HS on host cell surface plays a very important role in the process of many virus infections, and the affinity of the viral surface for HS may be a crucial determinant of tissue tropism, virus spread, or the establishment of latent infection. In most cases, the binding of the virus to HS seems to be relatively low-affinity, thus, HS may serve the purpose of concentrating the virus on the cell surface to facilitate the interaction with one or more high-affinity receptors, which induce endocytosis and subsequent cell membrane fusion [8,87,88,89].

HS also serves as an attachment factor in the host cell entry of many FLVs, including DENV, JEV, YFV, and TBEV. The efficiency of HS binding is virus-dependent. In multiple studies, HS has been shown to serve as a receptor or co-receptor for DENV infection of host cells [6,8,36,90]. Clinical strains of DENV1 can specifically interact with HS and heparin (HP). HP is the most negatively charged occurring natural polymer with an excellent anticoagulant activity. DENV2 binding to the two mutant cell lines of Chinese hamster ovary (CHO) cells defective in GAG expression was reduced by more than 75% compared to binding to wild-type CHO K1 [7]. Human endothelial cells express HSPGs that DENV4 specifically and efficiently interacts with in vitro. This was demonstrated by >60% reduction in DENV4 infection when human endothelial cells were pretreated with HP or HS [91]. The level of sulfation in GAGs on cell surfaces was found to be an important factor in success of in vitro JEV infection in both neurovirulent (RP-9) and attenuated (RP-2ms) strains [92]. In a separate study, both wild type (T1P1, CC27 and CJN) and laboratory-adapted strains (T1P1-L4, T1P1-S1, CJN-L1, CJN-S1, CC27-L1, CC27-L3, CC27-S6 and CC27-S8) of JEV infected BHK-21 and C6/36 cells by binding to surface HS [93]. An evident difference in attachment efficiency for JEV infection between CHO-K1 cells and its mutant with defects in GAG biosynthesis proved the importance of HS during JEV infection [94]. Recombinant TBEV with mutations in a defined genetic backbone showed HS-dependent phenotypes, resulting in an increased specific infectivity and binding affinity for BHK-21 cells and significant attenuation of neuro-invasiveness in adult mice [12]. Mutants of TBEV attached to HS-expressing cell lines with a 10-fold to 13-fold higher affinity than wild-type TBEV [95]. Although it was reported that mutations in the WNV E protein that enhanced binding to GAG molecules in vitro led to attenuation of virulence in a mouse model, the role of GAG in attachment of WNV is not clear [96]. A study conducted in our laboratory showed that ZIKV might also utilize GAGs to mediate host cell entry [13]. Surface plasmon resonance (SPR) kinetic measurements revealed that ZIKV E binds commercial HP strongly (K_D_ = 443 nM). Screening ability of ZIKV E binding to various natural GAGs with different disaccharide units, and varying length of HP oligosaccharides revealed that ZIKV E exhibits structural specificity of binding in addition to being electrostatically driven. The chondroitin sulfate (CS) GAG, isolated from human placenta tissues, showed comparable binding to ZIKV E (K_D_ = 658 nM) and it may be one of the candidate receptors for ZIKV. Main driving force of FLV-GAG interaction is electrostatically driven between surface basic and acidic residues on the FLVE and concentrated negative charge density on the sulfated polysaccharide chain [13,86,97]. Along the HS chain, relatively rigid, highly *N*-sulfo group rich domains (NS domains) of approximately 12–20 residues are adjacent to relatively flexible *N*-acetyl group rich domains (NA domains). This domain organization influences the orientation of the sulfate residues in space, and facilitates protein interactions with the sulfate residue [86]. Degree of sulfation in GAGs influences binding avidity and infection rate of FLVs shown in cell based studies. Treatment of BHK-21 target cells by a potent sulfation inhibitor, sodium chlorate, greatly reduced JEV binding avidity and infection [92]. When TBEV mutants with high HS affinity were incubated with sulfate-depleted BHK-21 cells, the growth was significantly delayed by the inhibition of sulfation [12]. HP and highly sulfated HS substantially inhibit DENV infection on Vero cells, with 99% and 87% inhibition respectively at the highest doses used, whereas the low-sulfate form of HS had no significant effect [6].

Both basic and acidic surface residues that contribute to hydrogen bonding play an important role in GAG-protein interactions in terms of both binding avidity and specificity [68]. Positively charged amino acids such Arg, Lys and His confer the envelope E protein to bind with negative-charged GAG. The GAG binding sites within FLVEs can be tentatively identified the crystal structures of FLVs [51,52,54,55,56]. Three basic residues, K305, K307, and K310, at a lateral ridge of domain III were speculated to form a potential GAG binding motif within DENV2 E [6]. Using an enzyme-linked immunosorbent assay (ELISA)-based GAG-binding assay, cell-based binding analysis and antiviral-activity assays, two critical residues, K291 and K295, in domain III were identified in GAG interaction with DENV E [61]. The highly DENV- and JEV-serogroup conserved K or R basic residues at positions 282, 284, and/or 288 in the domain I-HS-binding cluster are essential for virus function in both C6/36 and Vero cells [98]. Point mutations at E-138 and E-306 on the envelope protein of JEV may alter its ability to bind to HS on the cell surface and consequently change JEV infectivity, indicating these two sites are rather likely to be involved in determining efficiencies of JEV attachment, penetration, and eventual infection [94].

#### 2.2.2. Glycan-Mediated Dendritic Cell-Specific Intercellular Adhesion Molecule-Grabbing Non-Integrin Binding to Flavivirus

DC-SIGN, a carbohydrate binding, C-type, lectin-like molecule, is abundant in immature dendritic cells and is involved in the interaction with viruses, being an ancillary receptor [27,99]. DC-SIGN is composed of four domains, including a cytoplasmic domain, a transmembrane domain, an extracellular neck domain, and a carbohydrate recognition domain (CRD) [27,100].

Like other C-type lectins, DC-SIGN can recognize the glycosylation sites on the envelope FLVEs, including DENV E and WNV E (Table 1), thus facilitating the virus infection to host cells. DC-SIGN is a very important receptor for DENV E. Although Man receptor expressed on macrophages is another carbohydrate-binding receptor for DENV DC-SIGN is abundant in dendritic cells (DC) on human skin which is the first target for DENV once the infected mosquitos bite [21,101]. All four DENV serotypes derived from mosquito and mammalian cells infected DC-SIGN-expressing human monocytic cell line U937 cells with similar efficiency [44]. DC-SIGN transfected THP-1 monocytic cells also render these cells permissive to infection with all DENV serotypes [22]. DENV cannot bind nor infect the human B-cell line Raji, whereas DENV productively infects DC-SIGN transfected Raji cells [102]. Glycosylated WNV strains L1 infected DC-SIGN expressing THP-1 monocytic cell lines more efficiently than DC-SIGN negative cells [103]. Comparison of DC-SIGN and DC-SIGNR (a DC-SIGN related molecule, expressed on microvascular endothelial cells) in viral infection has been reported for WNV and JEV. DC-SIGNR promoted WNV infection much more efficiently than did DC-SIGN, particularly when the virus was grown in human cell types [28]. Growth of JEV derived from pig in DC-SIGNR expressing Daudi cells was greater than in DC-SIGN expressing Daudi cells [104]. ZIKV was also reported to infect DC-SIGN expressing HEK293T cells [40]. In contrast to DENV, WNV, and JEV, YF17D can infect immature and mature human DCs, but is independent of DC-SIGN [105]. It seems that YF17D interacts with DCs via different mechanism rather than DC-SIGN binding.

The interaction between DC-SIGN and the glycans of E protein on virus surface are essential for virus infection. The CRD domain in DC-SIGN C-terminal is responsible for responsible for recognition of high-Man glycans in the presence of Ca^2+^ (Figure 3) [46,104,106,107]. Molecular docking analysis between carbohydrates on DENV-2 virions and DC-SIGN revealed that hydrogen bonding and hydrophobic interactions are two of the forces that exist between glycan receptors and DC-SIGN, and most of the persistent H-bonds were formed from hydroxyl groups on Asn36, Glu366, Ser363 and Man [72]. The structural organization of *N*-linked glycans on the surface of E protein can favor the engagement of multiple E proteins dimers by each tetrameric lectin, enhancing the interaction between oligomeric DC-SIGN and virus [20]. The affinity of CRD to *N*-linked glycans on viral E proteins is dependent on the glycosylation level, increasing with the number of Man residues in the glycan [107,108]. Treatment of JEV particles with endoglycosidase H, which removes only *N*-linked sugars containing more than three terminal Man residues [109], or use of de-glycosylated JEV mutants reduced JEV infection of DC-SIGN-expressing cells [77]. DC-SIGN preferentially recognizes mannosylated E protein, rather than E protein with complex glycosylation [20]. DENV infection inhibited by Concanavalin A supported that α-Man carbohydrate residues participate in the attachment of DENV to DC-SIGN [21]. The involvement of Man during the interaction between JEV and DC-SIGN is further supported by the fact that JEV infection is significantly reduced in DC-SIGN expressing Raji cells in the presence of mannan, a competitor of insect-derived glycans [77].

## 3. Combating Flaviviruses with Carbohydrate-Based Or -Targeting Compounds

Extensive research has been performed to eliminate FLV entry at every step of host cell invasion including adsorption, membrane fusion, polypeptide processing, and viral assembly (Figure 2). The majority of the FLV inhibitors tested are comprised of carbohydrates, proteins, peptides, and small molecules, which are described in some excellent reviews [110,111,112,113]. The current review focuses on the inhibitors that are carbohydrate-based or carbohydrate-targeting (both viral and host cell glycan targeting). First, we examine the compounds that block the interactions between the host glycan, GAGs, and the viral envelope protein either by mimicking structure and activity of GAGs or binding to GAGs. Next, we investigate the compounds that block the interactions between viral glycan (*N*-linked glycan on FLVEs) and host receptor DC-SIGN by mimicking, attaching to, or permanently modifying the viral glycan on its envelope protein. Finally, we revisit the obstacles that were encountered when these compounds were studied and explore prospective strategies to eradicate FLVs infections.

### 3.1. Glycan-Based Entry Inhibitors Targeting Host Cell Glycans by Mimicking Structures and Activity of GAGs

Anionic surface GAGs provide an excellent starting point for preventing the initial attachment of FLV to the surface of host cells. HS is a universal eukaryotic surface GAG that all pathogenic FLVs bind to and, thus, various natural and synthetic HS mimetics have tested for their ability to inhibit GAG-FLVE interactions (Table 2).

#### 3.1.1. Natural and Synthetic GAGs

A previous study in our laboratory, we screened small polyanionic drugs, larger GAGs and their semisynthetic derivatives for their binding affinity to DENV2 E to investigate their structure-activity relationships [114]. Small polyanionic drugs (having two to six sugars), such as sucrose octasulfate, sulfated lactobionic acid, and sulfated β-cyclodextrin, failed to efficiently bind to DENV2 E despite their high level of charge density. This is presumably due to inability to efficiently occupy the entire putative GAG binding sites within DENV2 E. However, suramin, synthetic sulfonated aromatic, was able to bind to DENV2 E at comparable affinity, 40 nM, to that of HP, 15 nM. Next, we screened persulfated GAGs and HA (hyaluronan) oligosaccharides (decasaccharide to eicosisaccharide) where every hydroxyl group was sulfated. Binding affinity of persulfated GAGs, 4–15 nM, was similar or greater than that of HP whereas HA oligosaccharides showed size dependent binding, 57–100 nM. Using SPR, more rapid on-rate and off-rate were observed in HP and suramin than those of persulfated GAGs due to higher level of conformational flexibility HP and suramin possessed. Structure-activity relationships that promoted effective interactions between DENV2 E and polyanions were concluded: (1) minimum size 39 ‎Å; (2) high charge density; and (3) high level of structural flexibility. In a separate study, we also demonstrated inhibition of DENV2 infection using highly sulfated HS, HP, and suramin in vitro model involving Vero cells [6]. In our recent study, we concluded similar requirements for the structure-activity relationships between ZIKV E and natural GAGs and HP oligosaccharides, however HP bound ZIKV E with lower avidity, 443 nM, potentially due to less basic surface residue observed in GAG binding sites within ZIKV E [13]. Germi et al. reported that HP, but not CS-type B, inhibited binding of DENV2 and YFV in Vero cells [7]. Varying concentrations of HP (0.2 to 200 µg/mL) also inhibited YFV infection in Vero cells up to 97% with an ID_50_ of about 0.2 μg/mL. Despite having same degree of sulfation, CS-type E (CSE), but not CS-type D (CSD), was found to inhibit DENV infection in Vero cells [115]. Interestingly, CSE showed comparable inhibition of all serotypes of DENV and JEV infection to HP in Vero cells. The EC_50_ for the inhibition of DENV1-4 infection was 0.5–1.89 and 0.3–3.80 μg/mL for HP and CSE, respectively. EC_50_ of JEV infection inhibition by HP and CSE were 0.77 and 0.93 μg/mL, respectively. HS, CS-type A and B showed 50–70% inhibition activity against DENV4 in human endothelial cells at high concentrations (>10 µg/mL) whereas HP inhibited by >60% even at low concentration (≥1 µg/mL) [91]. These concentrations of GAGs can be easily achieved in plasma without toxicity.

#### 3.1.2. Fucoidan

Fucoidan, a natural polysaccharide from the marine algae, *Cladosiphon okamuranus*, is comprised of carbohydrate units containing glucuronic acid and sulfated Fuc residues [116]. Fucoidan was reported to selectively inhibit DENV2 infection, but not other serotypes, in BHK-21 cells. The IC_50_ against DENV2 by fucoidan was 4.7 μg/mL while those against DENV1, 3, and 4 ranged from 365 to greater than 1000 μg/mL. The inability of desulfated fucoidan, carboxyl-reduced fucoidan, and Fuc polymer (fucan) to inhibit DENV infection demonstrated that sulfation on the Fuc units and carboxyl group at the C6 of glucuronic acid were important for effective binding to DENV2 E. Structure analysis revealed that Arg 323 is also important for binding to DENV2 E. While fucoidans from many species of brown algae possess anticoagulant activity, those from *C. okamuranus* did not exhibit significant level of anticoagulant activity [117]. Thus, fucoidan from *C. okamuranus* makes an excellent natural polysaccharide candidate for selective inhibitor of DENV2 infection.

#### 3.1.3. Carrageenans

Talarico et al. tested the anti-FLV activity of sulfated polysaccharides, ĸ/ι/ν carrageenan G3d, from *Gymnogongrus griffithsiae* against all serotypes of DENV and reported these to be selective inhibitors of DENV2 infection in vitro models [118]. Carrageenans consist of linear chains of alternating (1→3)-β-d-Gal and (1→4)-α-d-Gal (or 3,6-anhydro-Gal). The IC_50_ of ĸ/ι/ν carrageenan against DENV1, 2, 3, and 4 infections were >50, 0.9, 13.9, and >50 μg/mL in Vero cells, respectively. The IC_50_ of ĸ/ι/ν carrageenan against DENV2 infection were 1.8 and 0.31 μg/mL in human hepatoma HepG2 and foreskin PH cells, respectively. In DENV3, IC_50_ was 10.4 and 9.5 μg/mL for HepG2 and PH cells, respectively. Surprisingly, neither ĸ/ι/ν carrageenan, HP, nor dextran sulfate 8000 could inhibit DENV infection even at the maximum concentration tested, 50 μg/mL, in C6/36 HT cells that are derived from *Aedes albopictus* mosquitoes that are main vector of DENV. In a subsequent study, ĸ/ι/ν carrageenans were used to test their inhibition against DENV2 infection in Vero and C6/36 HT cells [119,120]. All three carrageenans inhibited against DENV2 infection with ι-carrageenan being a most potent inhibitor (EC_50_ = 0.4 μg/mL) in Vero cells. However, only ι-carrageenan was able to inhibit DENV2 infection in C6/36 HT cells and at a 17.5-fold lower potency (EC_50_ = 7 μg/mL). The mode of action of ι-carrageenan differed in Vero and mosquito cells. Inhibition occurred at adsorption of DENV2 in Vero cells whereas it did not in mosquito cells. The order from the greatest degree of sulfation to the least per disaccharide unit follows: λ (3) > ι (2) > ĸ (1). It is interesting that ι-carrageenan demonstrated greatest inhibition against DENV2 infection even though ι-carrageenan had the greatest degree of sulfation. This reinforces that polyanion-DENV E interaction possesses structural specificity and is not entirely dependent upon electrostatic forces as found in our previous studies [6,114]. Carrageenans also have been reported to have anticoagulant activity and effects to enhance their activity has been employed by oversulfation and regioselective sulfation modification [121,122,123].

#### 3.1.4. Sulfated K5 Polysaccharides from *Escherichia coli*

K5 polysaccharides from *E. coli* and their chemically modified sulfated derivatives were evaluated for their anti-FLV activities against DENV2 [124]. K5 polysaccharides have the disaccharide unit of →4)-β-GlcA (1→4)-α-GlcNAc(1→, which is similar to de-sulfonated HS [125]. They were previously reported for their antiviral activity against human immunodeficiency virus (HIV), herpes simplex virus (HSV), human papillomavirus (HPV) and human cytomegalovirus (HCMV) [126,127,128,129]. Out of native form K5 and its sulfated derivatives (*N*-sulfo K5 (K5-NS), *O*-sulfo K5-OS (L) and *N*,*O*-sulfo K5-N,OS (L) with low degree of sulfation and K5 with high degree of sulfation K5-OS (H) and K5-N,OS (H)), K5-OS (H) and K5-N,OS (H) were most potent in inhibiting DENV2 infection in HMEC-1 and HMVEC-d. K5-OS (H) and K5-N,OS (H) inhibited DENV2 infection with EC_50_ of with an EC_50_ value of 113 and 111 nM in HMEC-1 cells, and 266 and 330 nM in HMVEC-d cells, respectively. HP, HS, CSA, and CSB were used as references and their EC_50_ against DENV2 infection in HMEC-1 cells were 77 nM (or 1 μg/mL), 6000 nM (60 μg/mL), 4200 nM (67 μg/mL), and 3300 nM (46 μg/mL), respectively. K5-OS (H) and K5-N,OS (H) present promising inhibitors against DENV2 because they not only have comparable level of inhibition of infection to HP, but K5 and its sulfated derivatives were evaluated to be devoid of anticoagulant activity [130].

#### 3.1.5. Curdlan Sulfate (Sulfated Glucan)

A sulfated 1R3-β-d-glucan, curdlan sulfate, showed excellent inhibition against infection of all serotype DENV and selectively against DENV2 infection (selectivity index > 1428) in various types of cell lines, such as BHK-21, C6/36, LLC-MK2, HL-60, and THP-1 [131]. The order of EC_50_ values in LLC-MK2 cells were as follows: DENV1 (262 μg/mL) > DENV4 (69 μg/mL) > DENV3 (10 μg/mL) > DENV2 (7 μg/mL). Curdlan sulfate was also reported as effective inhibitors of HIV and to exhibit anticoagulant activity [132,133].

#### 3.1.6. Sulfated Galactomannans

Chemically sulfated forms of galactomannans from seeds of *M. scabrella* (BRS) and *L. leucocephala* (LLS) demonstrated antiviral activity against DENV1 and YFV in vitro and in vivo models [134]. Galactomannans has a main chain of (1→4)-linked β-d-Man units substituted by α-d-Gal units with varying degree of d-Man units, which ranges from 60 to 80% [135,136]. Their structural analysis showed that galactomannans from BRS and LLS have Man to Gal ratio of 1:1 and 1:4, respectively. After sulfation, galactomannans from BRS and LLS had 0.62 and 0.5 degree of sulfation per unit and the average molecular weight of 620 kDa and 574 kDa. In C6/36 cells, concentration that reduced the YFV viral titer 100-fold in comparison to that of the positive control were 586 and 387 mg/L for BRS and LLS, respectively. In DENV1, these concentrations were 347 and 37 mg/L for BRS and LLS, respectively. At a dose of 49 mg/kg^−1^, BRS and LLS gave protection against death caused by YFV infection in 87.7 and 96.5% of young mice, respectively. In a separate study, chemically sulfated forms of galactomannans from fenugreek gum, guargum, tara gum, and locust bean gum were reported to for their antiviral activity against HIV and DENV2 [137]. Sulfated galactomannans generally had higher anticoagulant activity, 13.4–36.6 unit/mg, compared to that of dextran and curdlan sulfates, 22.7 and 10.0 unit/mg. They also exhibited similar anti-HIV and anti-DENV2 activities, 0.04–0.8 and 0.2–1.1 μg/mL, to those curdlan sulfates, 0.1 μg/mL, respectively.

#### 3.1.7. Sulfated Xylomannans

A diverse group of sulfated polysaccharides isolated from red, brown, and green seaweeds were tested for their anti-DENV activity [138]. Composition analysis revealed that the polysaccharides from *Grateloupia indica* (Gi) and *Gracilaria corticate* (Gc) were sulfated galactans, *Scinaia hatei* (Sh) gave sulfated xylomannans, *Stoechospermum marginatum* (Sm) and *Cystoseira indica* (Ci) sulfated fucans, and those from *Caulerpa racemose* (Cr) were heteropolysaccharides made up of Gal, Glc, arabinose (Ara) and xylose (Xyl). All sulfated polysaccharides most effectively inhibited against DENV2 in Vero cells with IC_50_ ranging from 0.12–20 μg/mL. Sulfated galactans (Gi), sulfated xylomannan (Sh), and sulfated fucan (Cr) had even greater anti-DENV2 activities with IC_50_ of 0.12–0.6 μg/mL compared to those of reference HP and dextran sulfate 8000 of 1.9 and 0.9 μg/mL. Sulfated polysaccharides from seaweeds have been generally reported to possess many biological activities including anticoagulant activity [139,140].

#### 3.1.8. Methyl-α-3-*O*-Sulfated Glucuronic Acid

A series of monomer carbohydrate compounds with varying conformations at anomeric center (α/β) and degree and position of sulfation on 1-methyl glucose (MeGlc), glucuronic acid (MeGlcA), and galactose (MeGal) were synthesize to evaluate their anti-DENV2 activities in BHK-21 cells [141]. The 3-*O*-sulfated MeGlcA was the most anti-DENV2 compound, inhibiting DENV2 infection by 87.5%, which is comparable to that of a reference compound sucrose octasulfate, 92.4%, at 500 µM. The EC_50_ of 3-*O*-sulfated MeGlcA was 120 µM, which was lower than that of sucrose octasulfate. Although lower than sucrose octasulfate, 3-*O*-sulfated MeGlcA still may not be a potent inhibitor. The chain length of sucrose octasulfate is only a disaccharide, which was not long enough to occupy the GAG binding site of DENV E in our previous study [6,114]. Thus, 3-*O*-sulfated MeGlcA being as a monosaccharide surely cannot efficiently bind DENV E to inhibit DENV infection. All the other derivatives with higher degree of sulfation also did not successfully inhibit DENV2 infection as expected. However, this study provides a structural rationale for designing low molecular weight sulfated carbohydrate compounds as anti-DENV agents. Two negatively charged groups a 3-*O*-sulfo and carboxyl group at C6 of GlcA were found important for anti-DENV activity. Carboxyl group at C6 of GlcA was also important in fucoidan [116]. No anticoagulant activity is expected at the monosaccharide length due to the importance of chain length for HP’s anticoagulant activity.

#### 3.1.9. Phosphomannopentaose Sulfate, Pentosan Polysulfate, and Suramin

Lee et al. evaluated the in vitro and in vivo anti-FLV activity of compounds that are currently approved or in clinical trials for DENV2 and encephalitis FLV, including phosphomannopentaose sulfate (PI-88), pentosan polysulfate (PPS), and suramin [142]. In BHK-21 cells, the EC_50_ against DENV2 infection followed the order from the highest to the lowest: PI-88 (200 μg/mL) > suramin (60 μg/mL) > PPS (30 μg/mL). However, EC_50_ against JEV infection followed the order: suramin (50 μg/mL) > PI-88 (40 μg/mL) > PPS (7 μg/mL). In both DENV2 and JEV, PPS was the most potent inhibitor in vitro models. The EC_50_ of PI-88 against WNV and MVE infections was 50 μg/mL. Surprisingly, PI-88 was the only compound with potent antiviral activity in vivo experiments. PI-88 inhibited against JEV, DENV2, and MEV infection in C57B1/6 mice at an acceptable toxicity concentration (0.5 mg/injection). In interferon-α and -γ receptor knockout mice, PI-88 increased the survival time from 15 to 22 days. This study is an excellent example of additional factors, such as physiochemical and pharmacological properties, alter the in vitro potency in vivo and must be carefully monitored. PI-88, PPS, and suramin have been reported to exhibit anticoagulant activities [143,144,145].

#### 3.1.10. Multivalent Lacto-*N*-Neotetraose Glycodendrimers

Aoki et al. identified that glycosphingolipid, neolactotetraosylceramide (nLc4Cer) was a host receptor for all serotypes of DENV in mammalian cells and that the non-reducing terminal disaccharide residue →4)Galβ(1→4)GlcNAcβ(1→ was critical for DENV2 binding [146]. Multivalent dendrimers containing lacto-*N*-neotetraose (nLc4, Galβ(1→4)GlcNAcβ(1→3)Gal β(1→4)Glcβ(1→) or lactose (Lac, Galβ(1→4)Glcβ(1→) in three different structures named Fan(0)3, Ball(0)4, and Dumbbell(1)6, were synthesized and tested for their anti-DENV2 activities in BHK-21 cells. At 500 μM concentration, the inhibition of DENV2 infection follows: Dumbbell(1)6-nLc4 (57%), Fan(0)3-nLc4 (44%), and Ball(0)4-nLc4 (32%). However, the same dendrimer structures synthesized with lactose did not show effective inhibition at this concentration. While this is not a HP/HS mimetic, it attempted to mimic the carbohydrate moiety of glycosphingolipid and amplify its binding to DENV by multivalency.

### 3.2. Non Glycan-Based GAG Binding Agents

Although these are not glycan based, peptides and proteins deserve attention for their anti-FLV activity resulting from specifically binding to cell surface GAGs.

#### 3.2.1. Lactoferrin

An antimicrobial protein, bovine lactoferrin, demonstrated anti-JEV activity by binding to cell surface HS in vitro [93]. Lactoferrin showed concentration-dependent inhibition against infection from HS-adapted JEV (CJN-S1) with IC_50_ of 25 μg/mL in BHK-21 cells. The inhibition was lower against infection from non-HS adapted JEV, reaching only 40% inhibition even at 200 μg/mL. HS binding of lactoferrin was determined by reduced anti-JEV activity from its binding to HP-Sepharose as well as the co-incubation with soluble HS. However, another potential pathway was discovered by the reduced anti-JEV activity when co-incubated with each low-density lipoprotein receptor and its antibodies.

#### 3.2.2. Basic Chemokine Derived Peptide

Basic peptides derived from antimicrobial chemokines, CXCL9 and CXCL12γ, with high affinity for GAGs have shown to inhibit DENV2 by its ability to bind to GAGs [147]. Using SPR, peptides derived from the carboxyl terminal regions of CXCL9 and CXCL12γ exhibited broad range of affinity to HP (K_D_ = 3.1–3559 nM). Inhibition of DENV2 infection in HMEC-1 cells by CXCL9 and CXCL12γ peptides ranged from EC_50_ of 11 to 48 μM with CXCL9 (74–103) being the most potent peptide. Using SPR competition assay, CXCL9 (74–103) blocked domain III of DENV2 envelope protein (DENV2 E) binding to immobilized HP even at 1:10 ratio for their concentrations in the sample.

### 3.3. Entry Inhibitors Targeting Viral Glycans

#### 3.3.1. Multivalent Mannose Glycodendrimers Mimicking Viral Envelope Protein Glycans

FLVs bind to a C type receptor DC-SIGN through the high-Man on their envelope proteins. Varga et al. synthesized multivalent Man glycodendrimers with varying valency to better compete with the high mannose expressed on the viral envelope protein to inhibit its interactions with DC-SIGN [148]. Surface plasmon resonance competition assay showed that the hexavalent glycodendrimer bearing six copies of bisamide gave the lowest IC_50_ of 6 μM to inhibit DC-SIGN binding to immobilized mannosylated bovine serum albumin. Interestingly, this same hexavalent glycodendrimer most successfully inhibited HIV and DENV2 infection in B-THP-1/DC-SIGN and Raji cells over-expressing DC-SIGN, respectively. Hexavalent glycodendrimer comparably inhibited against HIV and DENV2 infection with IC_50_ of 1 and 5.9 μM. This is an interesting approach to mimic the viral glycans, rather than the host glycans to compete for the carbohydrate-binding region on the host cell receptor.

#### 3.3.2. Lectin Based Inhibitors Targeting Viral Glycans

Rather than mimicking the viral glycans, several plant lectins were utilized to bind to the viral glycan on its envelope protein to block the viral envelope protein and DC-SIGN interactions. Three plant lectins, *Hippeastrum* hybrid agglutinin (HHA), *Galanthus nivalis* agglutinin (GNA) and *Urtica dioica* (UDA) isolated from the amaryllis, the snowdrop and the stinging nettle showed anti-DENV2 activities in vitro [149]. HHA specifically recognizes α1→3 and α1→6 Man residues, GNA recognizes α1→3 Man residues, and finally UDA recognizes GlcNAc residues. These lectins showed cell type dependent inhibition of DENV2 infection with EC_50_ of 0.1–2.2 and 4–56 µM in DC-SIGN transfected Raji cells and interleukin 4 (IL-4) treated monocytes, respectively. However, they did not exhibit any antiviral activities in Vero-B (DC-SIGN^−^). Both Raji/DC-SIGN^+^ and IL-4 treated monocytes express DC-SIGN on their cellular surface while Vero-B did not, which may explain the absence of anti-FLV activity in Vero-B cells.

Griffithsin (GRFT) is a lectin isolated from red algae *Griffithsia* and has been reported to exhibit antiviral activities against many enveloped viruses. It is 121 amino acid residues long, yet contains three distinctive carbohydrate-binding sites for Man and Glc [150]. GRFT successfully inhibited against JEV infection both in vitro and in vivo [151]. Pretreatment of GRFT in BHK-21 cells inhibited against JEV infection with IC_50_ of 265 ng/mL. In vivo data showed that *intraperitoneal* administration of GRFT (5 mg/kg) into BALB/c mice completely prevented mortality of JEV infection with a lethal dose. In a separate study, it was postulated that GRFT inhibits against JEV infection by binding to the viral glycan on the envelope protein [152].

#### 3.3.3. α-Glucosidase Inhibitors: Disrupting Proper *N*-Linked Glycosylation of Viral Glycoproteins

**Figure 4:**
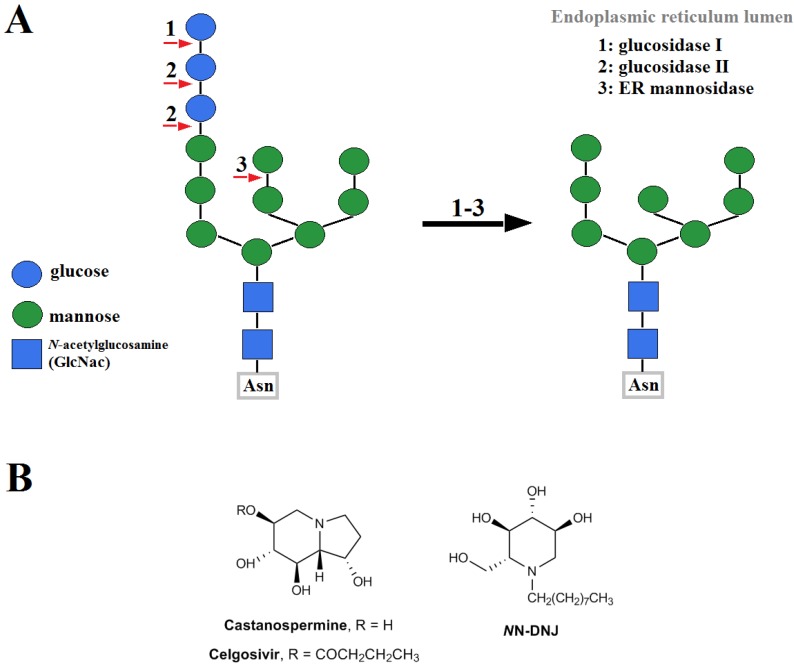
(**A**) Schematic of glucose- and mannose-removal by glucosidase I, II, and endoplasmic reticulum (ER) mannosidase during asparagine (Asn)-linked glycosylation in the ER lumen; (**B**) Chemical structures of α-glucosidase inhibitors, castanospermine, celgosivir, and *N*-nonyldeoxynojirimycin (*N*N-DNJ).

While 3.2.1 and 3.2.2 deal with competitive inhibitors to successfully interrupt viral glycan on the envelope protein and DC-SIGN in the extracellular space, glucosidase inhibitors permanently modify the “original” glycan structure of viral glycan in cytoplasm. Glucosidases first must properly trim the terminal glycans on the lumen of endoplasmic reticulum (ER) before sending the proteins over to Golgi apparatus for further processing. FLVs have three main glycoproteins: prM/M (premembrane/membrane protein), E (envelope protein), and NS1 (nonstructural protein 1). Improper *N*-glycosylation of viral glycoproteins can interrupt proper folding of prM, leading to destabilization of the prM/E complex, and improper viral particle assembly [153]. Iminosugar derivatives, such as castanospermine (CST), celgosivir (6-*O*-butanoyl-CST), and *N*-nonyldeoxynojirimycin (*N*N-DNJ), are some of the most well studied glucosidase inhibitors in FLV infection. The pathway of *N*-glycosylation and chemical structures of these inhibitors are shown in Figure 4.

#### 3.3.4. Castanospermine

Whitby et al. tested antiviral activity of castanospermine (CST) against all four types of DENV, YFV, and WNV in vitro and in vivo [154]. CST showed comparable inhibition level against all serotypes of DENV in Huh-7 and BHK-21 cells, however this activity was cell type dependent. For example, the IC_50_ against DENV2 infection was 85.7 and 1 µM in Huh-7 and BHK-21 cells, respectively. CST also showed inhibition against YFV by 57 and 93% at 50 and 500 µM. However, CST did not show significant inhibition against WNV even at high concentration (500 µM). In C57BL/6 and A/J mice, CST promoted survival at 10, 50, and 250 mg/kg of body weight per day. However, no protective effect was shown against WNV. It was postulated that the mechanism of inhibition was due to ineffective secretion of virus with improper *N*-glycosylation.

#### 3.3.5. Celgosivir (6-*O*-Butanoyl-CST)

Celgosivir is an oral prodrug of CST and inhibits α-glucosidase I and II, and its antiviral activities were tested in DENV [155,156,157]. Celgosivir effectively inhibited against DENV infections in BHK-21 cells with EC_50_ ranging from 0.16 to 0.68 µM depending on the serotype, which was partly due to accumulation of misfolded NS1 of DENV in the ER [156]. Interestingly, Celogosivir showed two-fold higher efficacy than that of CST when ADE mice were challenged with 50 mg/kg. However, a recent study demonstrated that celgosivir was not a potent inhibitor of ZIKV in Vero cells (EC_50_ was not determined even at 50 µM) [158]. Despite its great efficacy in vitro and in vivo, celgosivir did not succeed at treating DENV patients due to its rapid elimination [159].

#### 3.3.6. *N*-Nonyldeoxynojirimycin (*N*N-DNJ)

*N*-Nonyldeoxynojirimycin (*N*N-DNJ), a 9-carbon alkyl iminosugar derivative, was found to inhibit against DENV, JEV, and WNV [160,161]. Wu et al. reported that *N*N-DNJ inhibited more successfully against DENV than JEV in BHK-21 cells although the IC_50_ values were not reported.

In Institute of Cancer Research (ICR) mice, *N*N-DNJ successfully increased the survival rate by 40% against JEV infection, compared to the control group, at 200 mg/kg/day. Secretion of the E and NS1 proteins was greatly reduced, indicating that the proper *N*-glycosylation steps must be followed for successful virus assembly. In a separate study, *N*N-DNJ showed anti-WNV activity with IC_50_ of 4 µM in BHK cells [161].

## 4. Future Directions and Conclusion

A number of strategies might be used to improve GAGs and other sulfated polysaccharides to more effectively inhibit against FLV infections. The first classes of compounds studied were natural GAGs and then GAG mimetics that inhibit GAG-FLVE interactions. Although these compounds have the required level of charge density and structural flexibility to successfully occupy the GAG binding sites of FLV and inhibit against FLV infections in vitro, the majority of these also possess anticoagulant activities. Anticoagulant activity is problematic because it will promote plasma leakage in the patients infected with DENV and YFV, diseases that cause haemorrhagic fever. This undesired characteristic might be attenuated by processing these polysaccharides into smaller oligosaccharides that reach the minimum size required for occupying the E protein GAG-binding site (a HP decasaccharide corresponding to 39 ‎Å in the case of DENV). For example, HP is a very potent anticoagulant drug, however its anticoagulant activity was relatively low until it reaches a hexadecasaccharide [140,162,163]. Several different chain lengths of oligosaccharides must be prepared to retain anti-FLV activity while minimizing its anticoagulant activity. Sulfated polysaccharides can also go through chemical modifications to reduce their anticoagulant activity. In our laboratory, non-anticoagulant heparin (NACH) has been synthesized by periodate cleavage at the GlcA and IdoA residues located within and adjacent to the antithrombin III [164]. Upon chemical modification, NACH completely loses its anticoagulant activity. However, these structurally modified sulfated polysaccharides can also result in a reduced level of interactions to the GAG-binding sites of FLVE as shown in our interactions studies of DENV E and ZIKV E [6,13]. Multivalent glycopolymers or glycodendrimers can also be produced to amplify glycan-protein binding, as this is often how glycans and proteins are presented on the surface of cells in nature. Another potential pitfall of sulfated polysaccharides is their poor bioavailability. While exhibiting excellent in vitro antiviral activities, those activities of PPS and suramin did not translate well to in vivo experiments. The least potent inhibitor, PI-88, in vitro, showed the greatest efficacy, which was postulated to be caused by their interactions with other HP-binding proteins in vivo [142]. This drawback may be overcome by improved in vivo delivery approaches. The simplest approach would be to conjugate the sulfated polysaccharides to a molecule that has higher specificity towards the target such as envelope protein, or specific cell or organ types. There are antibodies against FLVE for research use, however no effective antibody is available for clinical treatment due to antibody dependent enhancement between different serotypes of DENV. DNA or RNA aptamers may be excellent alternatives to specifically target FLVE; they are cost efficient, nontoxic, non-immunogenic, and exhibit incredible specificity against their targets once the “hit” compound is found from a large library. Highly specific DNA based aptamers were discovered against FLVE in recent studies [165,166]. Conjugating sulfated polysaccharides with highly specific and synergistic compounds, such as DNA aptamers, may improve the potential hurdle of oral bioavailability. Lastly, an additional hurdle is presented in the treatment of pregnant women infected with ZIKV infection. Inhibitors against congenital ZIKV infection must be large enough and not readily metabolized in the blood stream so that they do not enter the cells and cross the placental barrier to avoid undesired additional harm to the fetus.

In conclusion, this review examined the role of carbohydrates in the pathogenesis of pathogenic FLVs and their antiviral inhibitors. First, we briefly introduced the general mechanism of host cell entry of FLVs. Then we described the glycosylation of FLV envelope protein, host surface GAGs, DC-SIGN, and interactions between them. FLV inhibitors that are both glycan-based and targeting were reviewed and strategies to improve carbohydrate-based inhibitors were discussed.

## Figures and Tables

**Table 1:** Dendritic cell-specific intercellular adhesion molecule-grabbing nonintegrin (DC-SIGN) are involved in receptor mediated host cell entry of many pathogenic flaviviruses.

Receptor	Virus	Cell	Reference
DC-SIGN	DENV-1, DENV-2, DENV-3, DENV-4	human monocytic cell line (U937)	[43]
DC-SIGN	DENV-2	Raji	[102]
DC-SIGN	DENV-1, DENV-2, DENV-3, DENV-4	HEK-293T, HeLa, Raji, monocyte-derived dendritic cell (MDDC)	[20]
DC-SIGN	DENV-1, DENV-2, DENV-3, DENV-4	THP-1	[22]
DC-SIGN	WNV	THP-1	[103]
DC-SIGN, DC-SIGNR	WNV	K562, MDDC	[28]
DC-SIGN, DC-SIGNR	JEV	Daudi	[104]
DC-SIGN	ZIKV	HEK293T	[40]

DENV: dengue virus; WNV: West Nile virus; JEV: Japanese encephalitis virus; ZIKV: Zika virus.

**Table 2:** Chemical and anti-flavivirus properties of heparin and heparan sulfate mimetics.

Compounds	Chemical Structures	Antiviral Activity				Anticoagulant Activity (Y/N)	References
		In Vitro	Flavivirus	Cell Type	In Vivo		
		(EC_50_ or IC_50_)					
Heparin	→4)-*N*-sulfo 6-*O*-sulfo-α-d-glucosamine (1→4)-2-*O*-sulfo α-l-iduronic acid(1→ per disaccharide unit	0.2 µg/mL	YFV	Vero		Y	[131]
		0.5–1.89 µg/mL, 0.77 µg/mL	DENV1-4, JEV	Vero			[115]
		1 µg/mL	DENV2	HMEC-1			[124]
CSE	β-d-glucuronic acid 1→3, *N*-acetyl, 4,6-di-*O*-sulfo β-d-galactosamine 1→4	0.3–3.8 µg/mL	DENV1-4	Vero		Y	[115]
		0.93 µg/mL	JEV				
Fucoidan	α-(1→3) linked fucose with sulfate groups substituted at the C-4 position on some of the fucose residues	4.7 µg/mL	DENV2	BHK-21		Generally, Y	[116]
	Alternating (1→3)-β-d-galactopyranoses and (1→4)-α-d-galactopyranoses (or 3,6-anhydrogalactopyranoses)	0.9 µg/mL	DENV2	Vero,		Y	[118]
Carrageenans		1.8–10.4 µg/mL		HepG2			
Kappa/iota/nu		0.31–9.5 µg/mL		PH			
		>50 µg/mL	DENV1-4	C6/36 HT (*Aedes albopictus* mosquito cells)			
iota		0.4 µg/mL	DENV2	Vero			[119],
		7 µg/mL		C6/36 HT			[120]
K5	4-β-glucuronyl-1,4-α-*N*-acetylglucosamine	113 µg/mL	DENV2	HMEC-1		N	[124]
K5-OS(H)		226 µg/mL		HMVEC-d			
K5-N,OS(H)		111 µg/mL		HMEC-1			
		330 µg/mL		HMVEC-d			
Curdlan sulfate (sulfated glucan)	branched β-d-(1→3) glucan backbone with piperidine-*N*-sulfonic acid	262 µg/mL	DENV1	LLC-MK2		Y	[131]
		7 µg/mL	DENV2				
		10 µg/mL	DENV3				
		69 µg/mL	DENV4				
Sulfated galactomannans	(1→4)-linked β-d-mannopyranosyl units substituted by α-d-galactopyranosyl units.	586 mg/L (BRS)	YFV	C6/36	Swiss mice, 87.7 and 96.5% protection at 48 mg/kg of animal weight.	Y	[134]
		387 mg/L (LLS)					
	*M. scabrella* (BRS): 1:1 mannose to galactose and *L. leucocephala* (LLS): 1:4	347 mg/L (BRS)	DENV1				
		37 mg/L (LLS)					
Sulfated polysaccharides from red, green, and brown seaweeds	Sulfated galactans, xylomannans, fucans, and heteropolysaccharides	0.12–20 µg/mL	DENV2	Vero		Y	[138]
Methyl-α-3-*O*-sulfated glucuronic acid	Methyl-α-3-*O*-sulfated glucuronic acid	120 µM	DENV2	BHK-21		N	[141]
PI-88 (phosphomannopentaose sulfate)		200 µg/mL	DENV2	BHK-21	Increased survival time from 15 to 22 days in C58B1/6 mice.	Y	[142]
	A mixture of highly sulfated, monophosphorylated mannose oligosaccharides	40 µg/mL	JEV				
PPS (pentosan polysulfate)	(1→4)-β-Xylan 2,3-bis (hydrogen sulfate) with a 4 *O*-methyl-α-d-glucuronate), this for PPS	60 µg/mL	DENV2			Y	
		7 µg/mL	JEV				
Suramin	8,8’-[carbonylbis[imino-3,1-phenylenecarbonylimino(4-methyl-3,1-phenylene)carbonylimino]] bis-1,3,5-naphthalenetrisulfonic acid	30 µg/mL	DENV2			Y	
		50 µg/mL					

BHK-21: baby hamster kidney fibroblasts; BRS: Sulfated galactomannans from M. scabrella; CSE: chondroitin sulfate type E; DENV1-4: dengue virus serotypes 1-4; EC_50_: half maximal effective concentration; HepG2: human liver cancer cell line; HMEC-1: immotalized human dermal microvascular endothelial cell line; IC_50_: half maximal inhibitory concentration; JEV: Japanese encephalitis virus; LLS: Sulfated galactomannans from L. leucocephala; YFV: yellow fever virus.
